# High dose antipsychotic therapy (HDAT) in the Greater Manchester mental health adult psychiatric inpatient setting

**DOI:** 10.1192/bjo.2021.312

**Published:** 2021-06-18

**Authors:** Oli Sparasci, Emma Horrell, Gemma Buston, Oliver Edge, Tatiana Campo Celaya, Daniel Shaw, Daisy Alston, Matthew Miller

**Affiliations:** 1 University of Manchester, Greater Manchester Mental Health NHS Foundation Trust; 2 Greater Manchester Mental Health NHS Foundation Trust

## Abstract

**Aims:**

To identify the number of adult inpatients prescribed HDAT across GMMH.

To establish whether guidelines for the prescribing and monitoring of HDAT are adhered to.

To consider the initiation of HDAT, evaluating whether prescriptions of HDAT are intentionally made by consultant psychiatrists and the MDT, or by rotational junior doctors.

**Background:**

High Dose Antipsychotic Therapy (HDAT) is defined by the Royal College of Psychiatrists as either: a total daily dose of a single antipsychotic which exceeds the upper limit stated in the BNF or A total daily dose of two or more antipsychotics which exceeds the BNF maximum as calculated by percentage.

The decision to prescribe HDAT should be made by a consultant psychiatrist and discussed with the patient and wider MDT. Clear documentation of this discussion, including the clinical indication, should be recorded within the case notes.

The use of HDAT comes with greater risk of physical health complications and requires regular monitoring of ECG, BMI and blood biochemistry. For patients detained under the Mental Health Act, consent and appropriate consultation with a SOAD should be sought for HDAT where the patient lacks capacity.

This audit investigates prescription of HDAT in the acute adult inpatient population within Greater Manchester Mental Health NHS Foundation Trust (GMMH).

**Method:**

Six junior doctors were recruited to collect data across the 5 sites covering general adult inpatients within GMMH. Data were collected week beginning 21st January 2020. Data were collected from all 20 general adult inpatient wards within the trust. Medication cards for each patient on the electronic bed-state at 9am on the day of the audit were checked for HDAT prescription. Subsequently, data were collected from electronic notes of patients identified as being on HDAT. Data were collated and submitted to the audit lead for analysis.

**Result:**

31 patients were identified as being on HDAT, of those, 21 instances of HDAT were commenced during the patients MDT, although in only 2 of these cases was it noted that the medication prescribed would result in initiating HDAT. Of the remaining cases, 8 were prescribed by junior doctors and 2 were unclear. 15 out of 31 patients had an ECG within a month prior to commencing HDAT, of 24 patients on HDAT for longer than 3 months, only 5 had a repeat ECG within this time.

**Conclusion:**

Guidelines are not closely adhered to, there is clear and necessary scope for improvement.

